# Adjuvants and Antigen-Delivery Systems for Subunit Vaccines against Tuberculosis

**DOI:** 10.3390/vaccines9090972

**Published:** 2021-08-30

**Authors:** Abu Salim Mustafa

**Affiliations:** Department of Microbiology, Faculty of Medicine, Kuwait University, Safat 13110, Kuwait; abu.mustafa@ku.edu.kw; Tel.: +965-2463-6505

**Keywords:** tuberculosis, subunit vaccines, antigens, adjuvants, delivery systems

## Abstract

The only licensed vaccine against tuberculosis is BCG. However, BCG has failed to provide consistent protection against tuberculosis, especially pulmonary disease in adults. Furthermore, the use of BCG is contraindicated in immunocompromised subjects. The research towards the development of new vaccines against TB includes the use of *Mycobacterium tuberculosis* antigens as subunit vaccines. Such vaccines may be used either alone or in the prime-boost model in BCG-vaccinated people. However, the antigens for subunit vaccines require adjuvants and/or delivery systems to induce appropriate and protective immune responses against tuberculosis and other diseases. Articles published in this Special Issue have studied the pathogenesis of BCG in children and the use of BCG and recombinant BCG as potential vaccines against asthma. Furthermore, the use of different adjuvants and delivery systems in inducing the protective immune responses after immunization with subunit vaccines has been described.

Tuberculosis (TB) is a global infectious disease that has been known to humans since ancient times. Although, various steps to control TB have been undertaken by different governments, as well as the World Health Organization, TB remains among the top 10 diseases in the world [1]. According to the most recent estimates from the World Health Organization, 10 million people developed the active disease, and 1.4 million people died of TB in 2019 [2]. The only licensed vaccine currently used against TB is the bacillus Calmette Guerin (BCG), which was obtained upon the attenuation of the pathogenic *Mycobacterium bovis* after subculturing on artificial medium for 13 years (1908–1921) and 231 passages [3]. In 1974, the World Health Organization included BCG in its list of vaccines recommended for the Expanded Program on Immunization [4]. Currently, BCG is administered to more than 100 million neonates per year and provides about 50% protection against clinical TB in children [5]. However, BCG is a live vaccine, and its administration leads to infection in about 1:10,000 to 1:1,000,000 children [6]. In this Special Issue of *Vaccines*, invited experts have contributed articles related to the pathogenesis of BCG in neonates, the protective potential of BCG against asthma, and the use of mycobacterial antigens as subunit vaccines in inducing protective immune responses by using different adjuvants and delivery systems.

The article by Al Busaidi et al. reports the spectrum of BCG-induced disease among children in Oman [7]. They included all children of <14 years of age who had laboratory-confirmed BCG disease from January 2006 to December 2018. The results of the study showed that 9.2 cases of BCG disease occurred in 100,000 vaccinated neonates. Children with immunodeficiency and those presenting the symptoms within 6 months of vaccination had more severe and disseminated disease. The age of disease presentation and the severity of BCG diseases were significantly associated with immunodeficiency status [7].

The BCG vaccine has also been reported to protect against other mycobacterial diseases (leprosy and non-tuberculous mycobacterial infections) [8] and non-mycobacterial diseases, including allergic diseases, such as asthma, and different types of cancers [9,10,11]. Allergic asthma is characterized by the secretion of antigen-specific T helper 2 (Th2) cytokines (e.g., IL-5 and IL-13) in large quantities [12]. On the other hand, the cytokines IFN-γ and IL-10 secreted by Th1 and T regulatory (Treg) cells, respectively, are associated with protection against asthma [13]. In mice, injection with BCG has been demonstrated to suppresses allergen-induced airway eosinophilia (a hallmark of allergic asthma), along with decreased production of Th-2 cytokine IL-5 and shifting the immune balance towards Th-1 cytokines in bronchoalveolar lavage fluid [14]. It has been further shown that recombinant BCG secreting murine IL-18 (rBCG-mIL-18) further increases Th-1 cytokine production in mice [15]. In the ovalbumin-induced murine model of asthma, rBCG-mIL-18 protected mice better than wild-type BCG against allergen-induced eosinophilia and IL-5 production [16]. Furthermore, it has been shown in humans that, compared to wild-type BCG, a recombinant BCG strain secreting human IL-18 (rBCG-hIL-18) induced stronger IFN-γ production by T cells cocultured with dendritic cells (DCs) from healthy BCG-vaccinated subjects [17]. In the article published by Kowalewicz-Kulbat et al. in this Special Issue, the authors have shown that in the presence of a major allergen of house dust mites (Der p 1), BCG- and rBCG-hIL-18-pulsed monocyte-derived dendritic cells (MD-DCs) co-cultured with naïve T cells from allergic patients resulted in a decrease in IL-5 production compared to non-pulsed MD-DCs cultured in the presence of Der p 1 alone. [18]. Based on these results, the authors suggest that BCG, and especially rBCG-hIL-18, may have therapeutic value in reducing the exacerbated Th2 responses in patients with allergic asthma.

BCG has been used as a vaccine to protect against TB since 1921, but it has failed to provide consistent protection in different parts of the world, especially against pulmonary TB in adults [9]. Furthermore, BCG can cause disseminated disease in immunocompromised individuals [19]. Hence, more effective and safer vaccines are required to protect against TB. Among the approaches towards the development of new vaccines against TB is the use of *Mycobacterium tuberculosis* antigens as subunit vaccines [20]. Such vaccines may be used either alone as priming vaccines or in a prime-boost model in BCG-vaccinated people. However, the antigens for subunit vaccines will require adjuvants and/or delivery systems to induce appropriate and protective immune responses against TB [21,22].

In this Special Issue, the results of the adjuvant effects of the cytokine IL-7and transcriptional factors (Id3, Bcl6, Bach2, and Blimp1) in increasing the duration of the immune memory induced by tuberculosis subunit vaccines was studied in mice by Han et al. [23]. Groups of mice were immunized three times at two-week intervals with the *M. tuberculosis* subunit vaccine Mtb10.4-HspX (MH) plus ESAT6-Ag85B-MPT64_<190–198>_-Mtb8.4-Rv2626c (LT70), together with adeno-associated virus-mediated IL-7 or lentivirus-mediated transcriptional factor Id3, Bcl6, Bach2, and Blimp1. The vaccine-induced immune responses were evaluated at 25 weeks after the last immunization. The results showed that IL-7 plus the TB subunit vaccine induced the formation of long-lived memory T cells and increased the expression of *Id3*, *Bcl6*, and *bach2*, which are the three main transcription factors involved in the generation of long-lived memory T cells. The results of immunization with the transcriptional factors along with the subunit vaccines showed that Bcl6 and Id3 resulted in the increased production of antigen-specific antibodies and long-lived memory T cells. These results showed that IL-7 and transcriptional factors Id3 and Bcl6 acted as adjuvants in helping the TB subunit vaccine to induce long-term immune memory, which plays a role in providing protection against *M. tuberculosis* infection.

Many other adjuvants and delivery systems have been used to improve the protective immune responses induced by subunit vaccines against TB, which include bacterial and viral vectors, liposomes, nanoparticles, plasmid DNA, and edible-based strategies, etc. [24,25,26]. A large number of such vaccines have been tested in animal models of TB with encouraging results [24,25,26]. Some of these subunit vaccines using various antigens, adjuvants, and delivery systems have reached Phase I and/or Phase II clinical trials in humans (Table 1).

The subunit vaccines listed in Table 1 have been shown to be safe in humans and induce appropriate immune responses correlating with protective potential. 

In conclusion, the use of appropriate adjuvants and delivery systems for tuberculosis subunit vaccines has gone beyond the experiments in animals and several clinical trials in humans have been conducted with satisfactory outcomes. These results provide hope for new TB vaccines that may have a safety profile better than the currently used BCG vaccine. Furthermore, such subunit vaccines may be able to replace BCG or can be used as boosters in BCG-vaccinated subjects in prime-boost strategies.

## Figures and Tables

**Table 1:** Adjuvants and delivery systems used with subunit vaccines undergoing clinical trials in humans.

Subunit Vaccine Antigens	Adjuvant ^a^/Delivery System ^b^	Vaccine Designation	Reference
Rv2608, Rv3619c, Rv3620c, Rv1813	GLA-SE	ID93/GLA-SE	[27,28,29]
Ag85B, ESAT6	IC31	H1:IC31	[30,31]
Ag85B, ESAT6, Rv2660c	IC31	H56:IC31	[32,33]
Ag85B, TB10.4	IC31	H4:IC31/AERAS 404	[34,35,36]
Mtb32A, Mtb39A	AS01_E_	M72/ AS01_E_	[37,38]
Ag85b, ESAT6, CFP10	CpG and aluminum salt	AEC/BC02	[39]
Ag85B, ESAT6, CFP10	BCG	Gam TBvac	[36,40]
Ag85A, Ag85B, TB10.4	Ad35	AERAS-402	[41]
Ag85A	MVA	MVA85A	[42]
Ag85A	MVA, ChAdOx1	MVA85A, ChAdOx1 85A	[43]
Ag85A, ESAT6	Flu-04L	TB/Flu-04L	[36]

^a^ Adjuvants = GLA-SE, IC31, AS01_E_, CpG and aluminum salt. ^b^ Delivery system = BCG, Ad35, MVA, ChAdOx1, Flu-04L.
